# Precipitation and temperature regulate species diversity, plant coverage and aboveground biomass through opposing mechanisms in large-scale grasslands

**DOI:** 10.3389/fpls.2022.999636

**Published:** 2022-12-01

**Authors:** Zhenyu Yao, Yue Xin, Liu Yang, Liqing Zhao, Arshad Ali

**Affiliations:** ^1^ Inner Mongolia Key Laboratory of Grassland Ecology and School of Ecology and Environment, Inner Mongolia University, Hohhot, China; ^2^ Yinshanbeilu Grassland Eco-hydrological National Observation and Research Station, China Institute of Water Resources and Hydropower Research, Beijing, China; ^3^ Inner Mongolia Geological Exploration Institute of China Chemical Geology and Mine Bureau, Hohhot, China; ^4^ Forest Ecology Research Group, College of Life Sciences, Hebei University, Baoding, Hebei, China

**Keywords:** climate, ecological mechanisms, *Leymus chinensis* community, topography, biotic factors

## Abstract

**Introduction:**

Although the relationships between species diversity and aboveground biomass (AGB) are highly debated in grassland ecosystems, it is not well understood how climatic factors influence AGB directly and indirectly *via* plant coverage and species diversity in large-scale grasslands along a topographic gradient. In doing so, we hypothesized that climatic factors would regulate plant coverage, species diversity and AGB due to maintaining plant metabolic and ecological processes, but the relationship of plant coverage with AGB would be stronger than species diversity due to covering physical niche space.

**Methods:**

To test the proposed hypothesis, we collected data for calculations of species richness, evenness, plant coverage and AGB across 123 grassland sites (i.e., the mean of 3 plots in each site) dominated by *Leymus chinensis* in northern China. We used a structural equation model for linking the direct and indirect effects of topographic slope, mean annual precipitation and temperature on AGB *via* plant coverage, species richness, and evenness through multiple complex pathways.

**Results:**

We found that plant coverage increased AGB, but species evenness declined AGB better than species richness. Topographic slope influenced AGB directly but not indirectly *via* plant coverage and species diversity, whereas temperature and precipitation increased with increasing topographic slope. Regarding opposing mechanisms, on the one hand, precipitation increased AGB directly and indirectly *via* plant coverage as compared to species richness and evenness. On the other hand, temperature declined AGB indirectly *via* plant coverage but increased *via* species evenness as compared to species richness, whereas the direct effect was negligible.

**Discussion:**

Our results show that niche complementarity and selection effects are jointly regulating AGB, but these processes are dependent on climatic factors. Plant coverage promoted the coexistence of species but depended greatly on precipitation and temperature. Our results highlight that precipitation and temperature are two key climatic drivers of species richness, evenness, plant coverage and AGB through complex direct and indirect pathways. Our study suggests that grasslands are sensitive to climate change, i.e., a decline in water availability and an increase in atmospheric heat. We argue that temperature and precipitation should be considered in grassland management for higher productivity in the context of both plant coverage and species diversity which underpin animals and human well-being.

## Introduction

**Figure 1:**
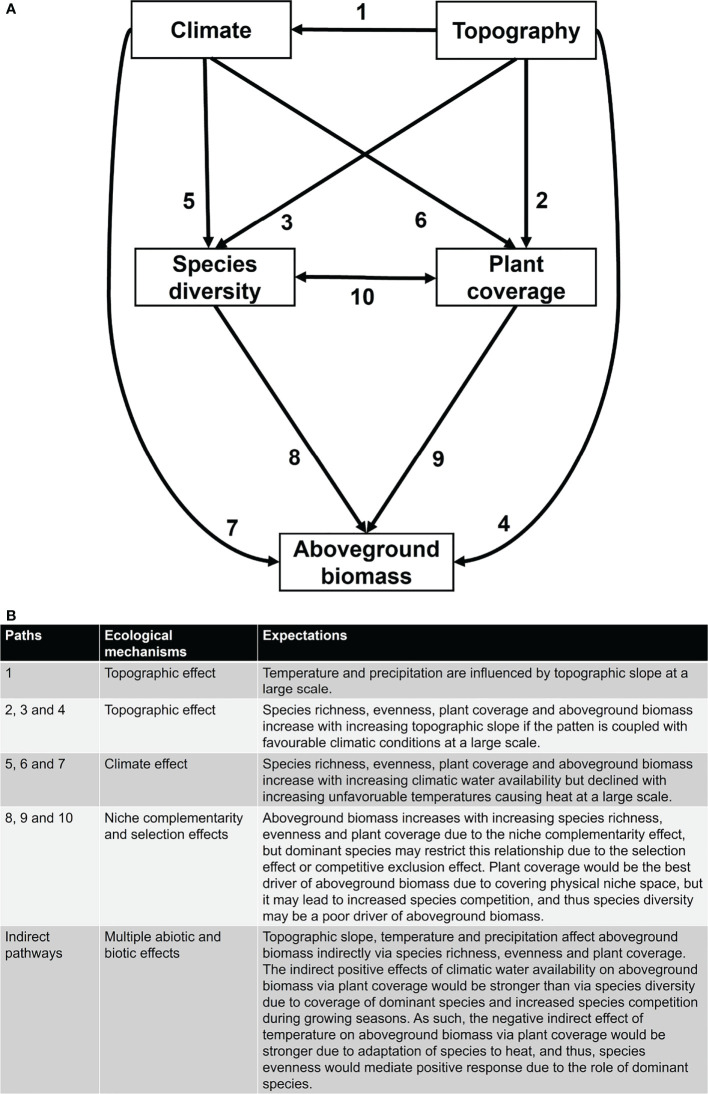
The brief conceptual model **(A)** and explanation of mechanisms and expectations **(B)** for elaborating the research questions and hypothesis in this study. Composite paths are labelled with numbering **(A)** for brief explanation **(B)**. Topography is represented by terrain slope, climate is represented by mean annual temperature (MAT) and mean annual precipitation (MAP), and species diversity is represented by species richness (S) and species evenness (J).

Understanding the divergent relationships (i.e., positive, negative and negligible) between biodiversity and aboveground biomass (AGB) has received much attention during the last few decades (Díaz et al., 2007; Grace et al., 2016). These divergent relationships are highly debated in grassland ecosystems and remain elusive (Gross, 2016; van der Plas, 2019). Nevertheless, climatic factors, such as precipitation and temperature, can greatly regulate biodiversity and AGB directly through plant metabolic, physiological and ecological processes, and such, as well as indirectly *via* species diversity and plant coverage through maintaining plant community assembly processes and species interactions in natural plant communities (Chu et al., 2016; Michaletz et al., 2018). However, few studies have focused on the role of plant coverage *versus* species diversity in regulating AGB in large-scale grasslands which are dominated by a specific plant species (Grace et al., 2016; Sanaei et al., 2018). Thus, our understanding is relatively unclear of how temperature and precipitation regulate AGB directly and indirectly *via* plant coverage and species diversity along a large-scale topographic gradient in natural grasslands (see a conceptual model in **Figure 1A**).

It is generally predicted that species diversity (either species richness, evenness or Shannon’s diversity) promotes AGB in grassland ecosystems due to the niche differentiation processes by component coexisting species (Tilman et al., 1996; Grace et al., 2016). There are two general ecological mechanisms, i.e., 1) the niche complementarity effect, and 2) the mass ratio or selection effect, which are usually put forward to explain variation in AGB which are underpinned by species (including functional trait and phylogenetic) diversity in grasslands (Diaz et al., 2007; van der Plas, 2019). The niche complementarity mechanism suggests that species with different niches are often able for more efficient use of available resources, thereby increasing AGB or productivity (Tilman et al., 1996; Loreau et al., 2001; Flombaum and Sala, 2008). For example, the presence of nitrogen-fixing legumes could promote other plant species and thus increase AGB (Hector et al., 2007; Marquard et al., 2009). The selection effect predicts that AGB is primarily determined by the characteristics of dominant or highly functioning species (Grime, 1998; Hector et al., 1999). For example, dominant species are considered to be more stable in the community and thus play an important role in regulating AGB whereas rare species play a negligible role (Sanaei and Ali, 2019). In most cases, studies have shown that the niche complementarity and selection effects may not act independently but jointly in explaining AGB in different plant communities (Diaz et al., 2007; Sanaei et al., 2018; van der Plas, 2019). Moreover, this situation is complex due to the strength, magnitude and direction of the relationship between species diversity and AGB as well as multiple indices of species diversity (Wilsey and Potvin, 2000; Polley et al., 2010). For example, it is always not true that species diversity increases AGB, and thus, negative and negligible relationships have also been reported in grasslands (van der Plas, 2019, and references therein). These divergent relationships between species diversity and AGB suggest that several internal (e.g., plant coverage) and external (e.g., topography and climate) factors are shaping species diversity and AGB in grasslands which needs further investigation across biomes for better understanding (**Figure 1**).

As compared to species diversity, plant coverage could be the best predictor of AGB due to physical coverage of the niche space which is in turn related to multiple ecological processes such as nutrient cycling, secondary productivity, and livestock feed (Marquard et al., 2009; Ji et al., 2009; Sanaei et al., 2018b, Grace et al., 2016). Although plant coverage is not the direct measure of biodiversity, it can greatly predict species richness, plant density and AGB due to the proportional physical use of a certain space by plants which can be directly and indirectly related to the niche space (Marquard et al., 2009). For example, plant communities with high plant coverage may use more environmental resources than communities with low plant coverage (Ji et al., 2009), and thus, plant coverage can increase species diversity and AGB (Sanaei et al., 2018). Moreover, high plant coverage may increase AGB directly and indirectly *via* species diversity through the coexisting of a diverse array of species having different plant coverage which could be able to use the available resources more efficiently, i.e., the mixture of shade-tolerant and shade-intolerant species (Yachi and Loreau, 2010; Schmid and Niklaus, 2017; Sanaei and Ali, 2019). However, the overruling effect of a few dominant species having high plant coverage may modulate the relationship between species diversity and AGB through the selection or competitive exclusion effect (Grime, 1998; Hector et al., 1999; Sanaei and Ali, 2019).

Beyond species diversity and plant coverage, climatic factors (e.g., mean annual temperature and mean annual precipitation), soil properties (e.g., soil nutrients and textures), and topographic factors (e.g., altitude and slope) are also known to be important direct and indirect drivers of AGB *via* its biotic factors such as species diversity and plant coverage (Chu et al., 2016; Grace et al., 2016; Sanaei et al., 2019; Cheng et al., 2021). For example, the amount of optimal precipitation can increase the length of the growing season through climatic water availability, and hence, plants can grow well under favorable climatic conditions, and this process promotes AGB directly and indirectly *via* species diversity and plant coverage (Chu et al., 2016; Poorter et al., 2017; Ali et al., 2019). However, the effects of mean annual temperature on plant diversity, coverage and AGB depend on climatic zones. For example, in cold regions, low temperature is the primary limiting factor for plant growth, and thus, an optimal increase in temperature could promote AGB (Heskel et al., 2016). However, in warmer regions, high temperatures may decline AGB due to changes in the plant metabolic processes (Kerkhoff et al., 2005). Likewise, topographic factors can influence AGB by regulating water availability and temperature (De Frenne et al., 2021). In middle and high latitudes, the topographic slope is an important factor in determining ecological conditions through the amount of solar radiation received by the ground, which can produce unique microclimates (Bennie et al., 2008). As such, environmental factors such as climatic water and temperature differ markedly at different altitudes (Sun et al., 2013) which in turn affect the availability of soil moisture and nutrients (Sanaei et al., 2019). Yet, we do not fully understand how temperature and precipitation influence AGB directly and indirectly *via* species diversity and plant coverage in large-scale grasslands (**Figure 1A**).

In this study, we examined the direct and indirect effects of topographic and climatic factors on AGB directly and indirectly *via* species richness, evenness and plant coverage in the natural grassland communities dominated by *Leymus chinensis* in northern China (**Figure 1A**). As the studied plant communities were dominated by *L. chinensis*, we, therefore, used species richness and species evenness as two independent drivers of AGB to represent the two aspects of species diversity while considering plant coverage as the physical coverage of vegetation. As such, we used topographic slope, mean annual precipitation and mean annual temperature as the main regulators of species diversity, plant coverage and AGB because the studied sites show substantial temperature and precipitation seasonality along topographic gradients. In doing so, we address the following research questions: 1) How do topographic slope, mean annual precipitation and mean annual temperature affect species richness, evenness, plant coverage and AGB in the grasslands dominated by *L*. *chinensis*; 2) what is the main mediating factor – species richness, evenness and/or plant coverage – for linking the response of AGB to mean annual precipitation and mean annual temperature along topographic gradient; and 3) what is the main ecological mechanism – the niche complementarity and/or selection effect – for explaining the relationships between species richness, evenness and AGB while considering plant coverage as a potential endogenous factor? We hypothesize that climatic factors regulate plant coverage, species diversity and AGB due to maintaining plant metabolic and ecological processes, but the relationship of plant coverage with AGB is stronger than species diversity due to covering physical niche space (see **Figure 1A** for a brief model, and **Figure 1B** for a brief explanation).

## Materials and methods

### Study area and grassland communities

**Figure 2:**
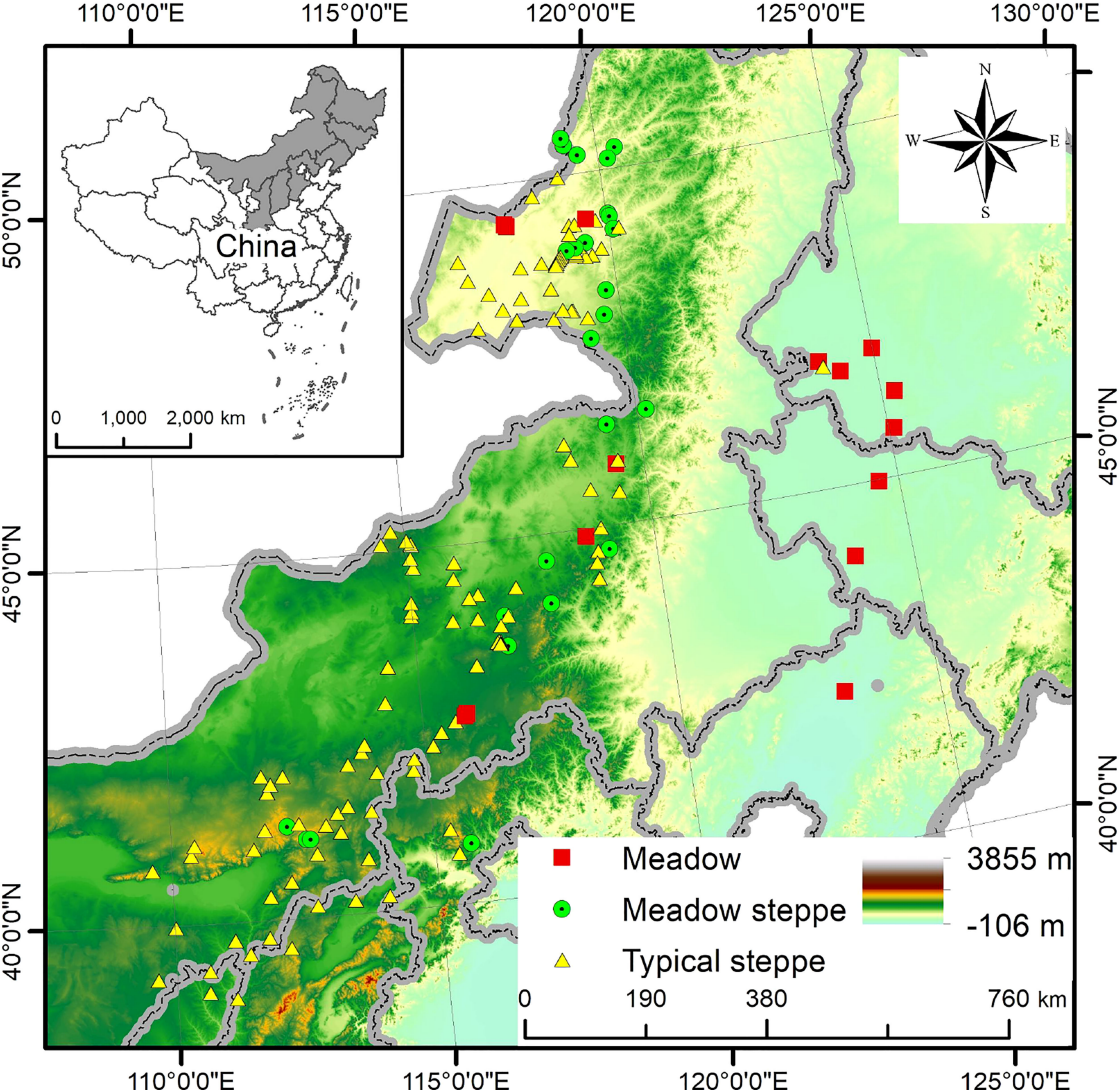
Map of the sampling sites of the grasslands dominated by *Leymus chinensis*. Different shapes and colors show three different community types dominated by *L*. *chinensis* in the study area.

The study was conducted in grassland communities, dominated by *L. chinensis* at a large scale, and the study sites are located between 109°37′—125°16′ E and 38°56′—50°31′ N, spanning the Loess Plateau, the Inner Mongolia Plateau and the northeast Plain in northern China (**Figure 2**). The altitude is high in the west and low in the east, dropping from 2023 m to 129 m. The region has a temperate climate with a mean annual temperature between -2.9 and 7.9°C. Mean annual precipitation varies greatly, with precipitation being mainly concentrated in the growing season. There is also a notable rainfall gradient spanning from 209 mm in the west to 498 mm in the east on average. The main soil types are chestnut, chernozem, and salinized meadow and loess soil.

Vegetation types from the west to east are desert steppe, typical steppe and meadow steppe where the meadow. *L. chinensis* communities are continuously distributed in southern Eastern Siberia, northern Mongolia, the middle and lower parts of the hilly area of the eastern and western foothills of China’s Great Khingan Mountains, and the Songnen Plain. In the central and western Mongolian Plateau and northern Loess Plateau, *L. chinensis* communities are largely distributed in relatively humid areas. Due to the wide distribution, there are several types of grassland communities across the region. Specifically, *L. chinensis* meadows are formed on the non-zonal soils (saline-alkali soil and meadow soil) in the northeast plain and within the eastern Greater Khingan Mountains. Meadow steppe is formed on zonal soils (chestnut soil or chernozem soil) in the west, whereas the Mongolian Plateau and the loess plateau to the west form a typical steppe (Zhu, 2004). These areas span semi-humid, semi-arid, and arid climatic zones. The plant growing seasons in the study area start in May and end in October.

### Data collection and quantification of variables used in the analyses

Field surveys were conducted during peak grass plant productivity from mid-July to late August during 2016-2020. We randomly selected those sites where *L. chinensis* was dominant. Then, three 1×1 m plots were established within each site, and thus, we sampled 369 plots in total across 123 sites. Within each plot, we recorded species composition, total coverage, density and height. We harvested and weighed the aboveground parts of the plants. For measuring AGB, samples were oven-dried to a constant mass at 65°C for 48 hours before weighing.

The total plant coverage of each species was recorded as the ratio of the vertical projection area of each species to the total area of the plot (Ji et al., 2009). Species richness was calculated by the observed number of species in each plot, whereas species evenness was calculated as the distribution of relative AGB across the species in a plot. These biotic calculations were conducted in the *vegan* package in R statistical software (Oksanen et al., 2015; R Development Core Team, 2019). We used mean data within each site, and thus, we used 123 sites in total.

A handheld Global Positioning System (GPS) was used to measure the geographical position and elevation of each sample site. The slope of each sample site was extracted using ArcGIS (10.5). To test the direct and indirect influences of climatic factors on plant coverage, species diversity and AGB, we extracted mean annual precipitation and mean annual temperature from the WorldClim database (https://www.worldclim.org/) at a resolution of 30 seconds (~1 km) (Fick and Hijmans, 2017).

Note that we used mean data for each biotic variable per site (**Table S1**), and thus, 123 sites were used in the statistical analyses for better representation of meta-sites to cover the gradient in abiotic and biotic factors. The list of observed species in the study region and their basic summary are provided in **Table S2**.

### Statistical analyses

The Kruskal-Wallis H test (also known as the “one-way ANOVA on ranks”) was used to determine if there were statistically significant differences in species richness, evenness, plant coverage and AGB as well as topographic and climatic variables among three types of grassland communities dominated by *L. chinensis*. To test the proposed research questions and hypothesis, we used a brief conceptual model (**Figure 1A**) with the following specific paths in structural equation modelling (SEM): 1) topographic slope affected mean annual precipitation, mean annual temperature, species richness, evenness, plant coverage and AGB directly; 2) mean annual precipitation and mean annual temperature affected species richness, evenness, plant coverage and AGB directly; 3) species richness, evenness and plant coverage affected AGB directly; and 4) species richness, evenness and plant coverage provided feedback to each other. We used cut-off criteria to evaluate the SEM fit to the meta-site data, which included the maximum likelihood chi-square (*χ^2^*) test and standardized root means square residual (SRMR) (Grace et al., 2016). The SEM was considered accepted if the *χ^2^* test statistic had *P* > 0.05 and SRMR < 0.08. We also considered the comparative fit index (CFI), which shows that results could be less affected by sample size if the value is greater than 0.95 and validated this calculation using the goodness-of-fit index (GFI), which suggests a good SEM fit if the value is greater than 0.95 (Hoyle, 2012). We preferred to use the SEM for testing the research questions and hypothesis as it allows us to integrate multiple factors in a single model structure, which is the best tool to test the multiple research questions and hypotheses in a single framework (Grace et al., 2016). We dropped the correlation path between mean annual precipitation and temperature to avoid the saturated or overfitted SEM, following the *χ^2^* test statistic with an associated *P*-value. The SEM was conducted using the *lavaan* package in R (Rosseel, 2012).

To complement the results from SEM, we also tested the linear regression models between tested variables by using a bivariate plot and Pearson’s correlation matrix. All data were standardized before analysis (standard deviation = 1, mean value = 0) to improve the linearity and normality as well as to compare the standardized effects of multiple predictors on response variables (Zuur et al., 2009; Grace et al., 2016; Ali et al., 2019).

## Results

**Figure 3:**
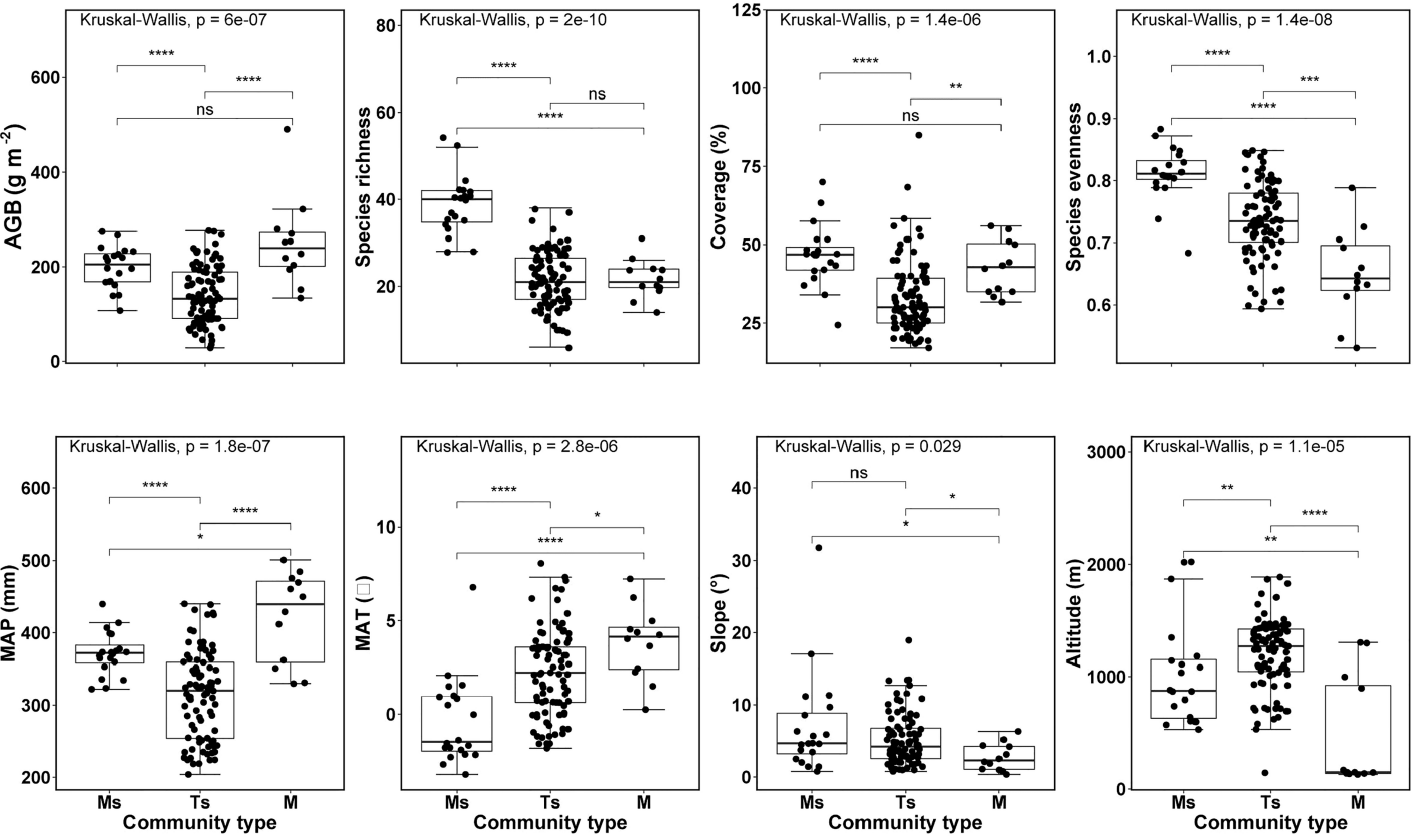
Differences for variables, used in the structural equation modelling, among three grassland community types dominated by *Leymus chinensis* in northern China. See **Tables S1**, **S2** for the summary. MAP, mean annual precipitation; MAT, mean annual temperature; and AGB, aboveground biomass; Ms, meadow steppe; Ts, typical steppe; M, meadow. p:****and ***<0.001, **<0.01, *<0.05, and ns >0.05.

The site maximum, minimum and mean (± SD) observed AGB values across all *L. chinensis* communities were 490.8g m^-2^, 28.5g m^-2^ and 160.9 (± 71.2) g m^-2^, respectively. The maximum, minimum and mean (± SD) observed species richness values were 49, 6 and 24 (± 9) species per site. Among biotic factors, AGB, species richness and plant coverage of the typical steppe community were the lowest and were significantly different from the other two communities. Species evenness was significantly different across three community types. As such, we found significant differences in topographic and climatic factors across three *L. chinensis* community types (**Figure 3**; **Table S1**).

**Figure 4:**
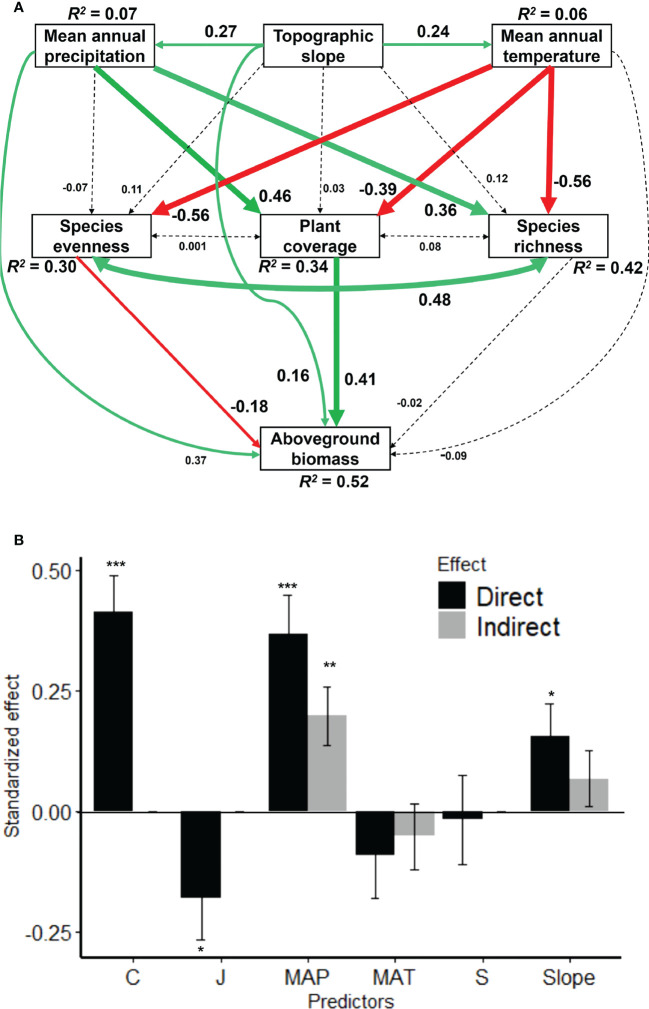
Structural equation model **(A)** for linking the direct and indirect effects of topographic slope and climatic factors (MAT and MAP) on aboveground biomass (AGB) *via* plant coverage, species richness, evenness in large-scale scale grasslands dominated by *Leymus chinensis* in northern China. Solid arrows represent significant (*P* < 0.05) effects and dashed arrows represent non-significant effects (*P* > 0.05). Red arrows represent negative effects while green arrows represent positive effects with a standardized value near each arrow. *R* ^2^ value associated with the response variable indicates the proportion of variation explained by predictors. Bar chart comparison of the direct and indirect effects of predictors on AGB **(B)**. See **Table S3** for the summary. Model-fit statistic: CFI = 0.999; GFI = 0.996; SRMR = 0.034; Chi-square test statistic = 1.344 with *P*-value = 0.248 and degrees of freedom = 1. Significance levels **(B)**: ***P < 0.001; **P < 0.01; *P < 0.05. MAP, mean annual precipitation; MAT, mean annual temperature; C, plant coverage; J, species evenness; S, species richness; and AGB, aboveground biomass.

**Figure 5:**
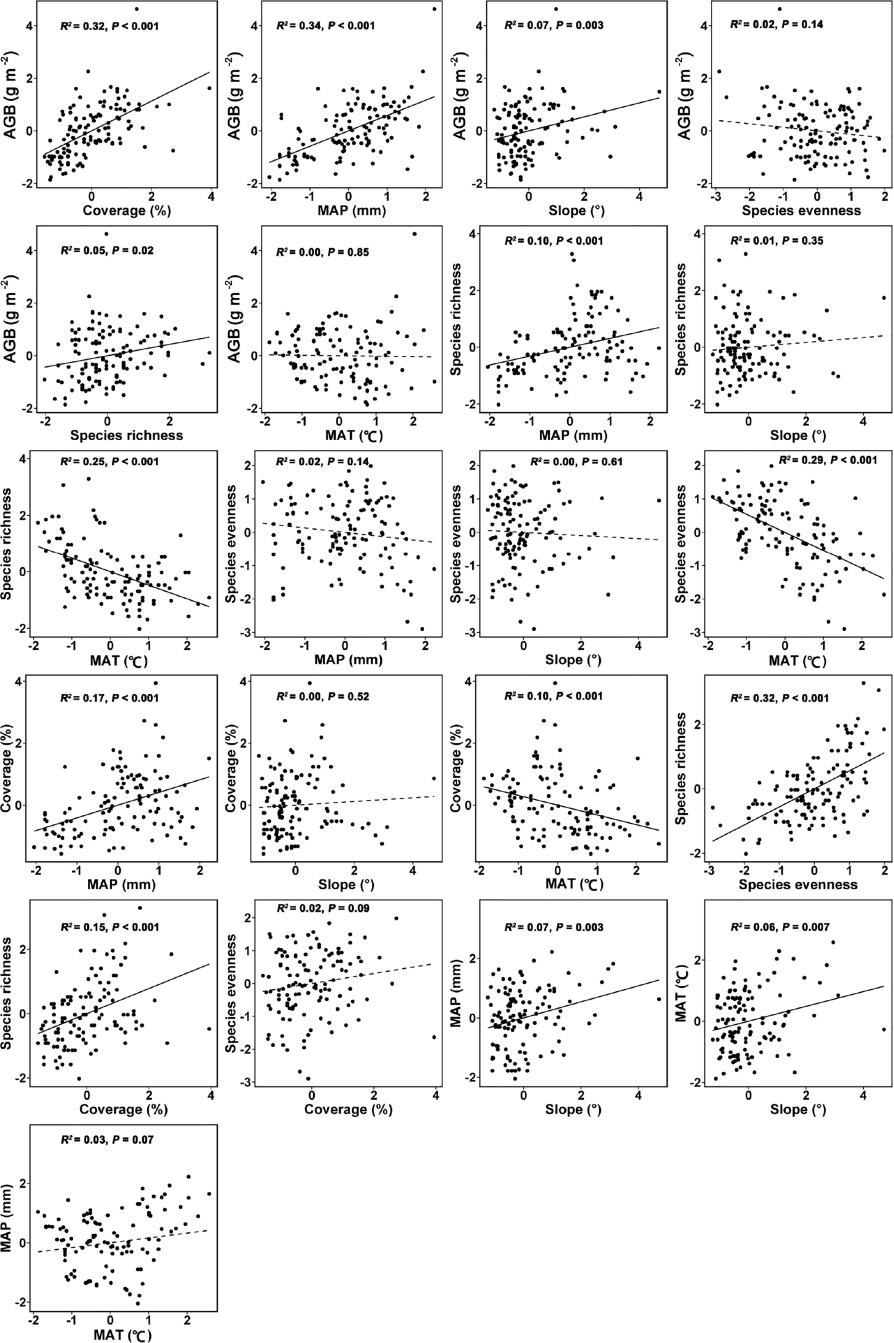
The bivariate relationships between tested variables used in the SEM for linking topographic slope, climatic factors (MAP and MAT), plant coverage, species richness, evenness and AGB of *Leymus chinensis* communities in northern China. Solid line represents a significant relationship whereas a dashed line represents a non-significant relationship. Abbreviations are explained in the caption of **Figure 4**.

The tested SEM explained 52%, 42%, 34% and 30% of the variations in AGB, species richness, plant coverage and species evenness, respectively (**Figure 4A**). Mean annual temperature and precipitation increased significantly with increasing topographic slope, and they were somehow positively correlated with each other (**Figure 5**, **S1**). However, topographic slope did not significantly alter species richness, plant coverage and species evenness, but imposed non-significant positive effects on them (**Figure 4A**). However, species richness and plant coverage increased significantly with increasing mean annual precipitation but declined with mean annual temperature. In partial contrast, species evenness declined significantly with mean annual temperature but declined non-significantly with mean annual precipitation (**Figure 4A**). As such, AGB increased significantly with increasing mean annual precipitation and topographic slope as compared to mean annual temperature. Importantly, AGB increased significantly with increasing plant coverage but declined with species evenness, whereas species richness possessed a non-significant negative effect on AGB. Species richness, evenness and plant coverage were positively correlated in SEM but this positive correlation was stronger between species richness and evenness (**Figure 4A**).

The comparison of direct and indirect effects (bar charts) showed that plant coverage was the top direct driver of AGB followed by mean annual precipitation, topographic slope, species evenness, species richness, and mean annual temperature (**Figure 4B**). Topographic slope increased AGB indirectly *via* mean annual precipitation, temperature and plant coverage but somehow declined *via* species richness and evenness, and thus, the indirect effect size was higher *via* precipitation (**Figure 4B**; **Table S3**). As such, precipitation increased AGB indirectly *via* plant coverage, species richness and evenness, and the indirect effect size was higher *via* plant coverage. In contrast, temperature declined AGB *via* plant coverage and species richness but increased *via* species evenness, and thus, the total indirect effect was negative (**Figure 4B**; **Table S3**).

The bivariate relationships (**Figure 5**) and Pearson’s correlation matrix (**Figure S1**) showed almost similar trends as shown by the paths of SEM (**Figure 4**).

## Discussion

Although the relationships between species diversity and AGB are highly debated in grassland ecology (Gross, 2016; van der Plas, 2019), we show how temperature and precipitation regulate plant coverage, species diversity and AGB in grasslands dominated by *L. chinensis* along topographic gradient in northern China. We found that mean annual precipitation and temperature controlled AGB directly and indirectly *via* plant coverage and species diversity through opposing mechanisms along increasing topographic slope. On the one hand, precipitation increased AGB directly and indirectly *via* plant coverage better than species richness and evenness, indicating the role of a longer length of growing season due to the water availability which is an important factor for plant physiological, metabolic and ecological processes (Gillman and Wright, 2014; Poorter et al., 2017; Ali et al., 2019; Cheng et al., 2021). On the other hand, mean annual temperature decreased AGB directly and indirectly *via* plant coverage better than species richness but increased *via* species evenness, indicating that plant coverage and species richness are sensitive to an increase in temperature (i.e., heat) but species evenness can maintain this adverse effect through the compensatory role (Isbell et al., 2015; Chu et al., 2016; Michaletz et al., 2018; Sullivan et al., 2020). By considering the divergent effects of precipitation and temperature, we found that plant coverage increased AGB better than species richness and evenness, indicating the role of occupancy of physical niche space (Marquard et al., 2009; Ji et al., 2009; Sanaei et al., 2018b).

We found that AGB, but somehow species richness, plant coverage and species evenness, were positively controlled by the topographic slope. However, it is noteworthy that the slope of the studied region is gentle, i.e., low slope (see **Table S1**), which is usually conducive to the growth of plants due to the higher availability of light and soil nutrients (Sanaei et al., 2018; Jucker et al., 2018). Moreover, the topographic slope can greatly control soil moisture and solar radiation, and thus, an increasing gentle slope can result in greater water availability which could promote biodiversity and AGB with high plant coverage (Sanaei et al., 2019; De Frenne et al., 2021). As such, we found that temperature and precipitation increased equally with increasing topographic slope, and they were somehow positive correlated with each other, which in turn influenced species diversity, plant coverage and AGB through opposing mechanisms.

On the one hand, we found that mean annual precipitation promoted AGB directly and indirectly *via* plant coverage, but not *via* species richness and evenness, in the studied grasslands. This is possibly due to the reason that rain events provide enough soil moisture and maintain high climatic water availability (Gillman and Wright, 2014; Poorter et al., 2017; Ali et al., 2019; Cheng et al., 2021). In arid and semiarid regions, climatic and soil water availability is typically a limiting factor for plant growth, and thus, available moisture generally promotes plant coverage and AGB as compared to species diversity due to the species’ competition for available resources (Butterfield, 2015; Berdugo et al., 2019). The plant coverage and AGB stock of perennial plant species is the result of photosynthesis which depends on water availability, and thus, high water availability can promote carbon sequestration *via* high plant coverage, thereby increasing AGB in plants (Kerkhoff et al., 2005; Yang et al., 2019; Cabon et al., 2022). In addition, the water use efficiency of plant species is generally influenced by the depth of the root system, and thus, grasses have higher water use efficiency because their root systems are shallow and have many fibrous roots (Nippert and Knapp, 2007). Through this understanding, grasses can efficiently use surface water for growth and to maintain plant coverage and AGB. As such, our studied grasslands were dominated by *L. chinensis* which is a perennial tall and erect grass, as most of the grasses occupy the top layers of the studied grassland communities. In sum, our results warn that drought (i.e., a decline in water availability) can greatly decline AGB directly and indirectly *via* plant coverage in the studied grasslands (Trenberth et al., 2014).

On the other hand, we found that mean annual temperature did not regulate AGB directly but did regulate indirectly *via* species evenness and plant coverage through opposing effects. Through declined species evenness, temperature increased AGB indirectly *via* adjusting plant metabolic and physiological processes of dominant resistive species to temperature sensitivity. The increase in temperature can promote the metabolism of certain adaptive plants to high temperatures which could prolong the growth cycle of plants, thereby increasing the AGB of a community (Gillman and Wright, 2014; Sullivan et al., 2020). However, we found that temperature declined plant coverage and species richness as well and that the indirect effect of temperature on AGB *via* plant coverage than species richness was negative. This result indicates that certain adaptive plant species can occupy the physical niche space, which could lead to less AGB due to the selection effect as compared to the niche complementarity effect (Loreau et al., 2001; Heskel et al., 2016). In sum, our result shows that temperature regulates the metabolic, physiological and ecological processes of certain species with uneven distribution of plant coverage, which in turn leads to less AGB indirectly. The overall negative indirect effect of temperature on AGB *via* plant coverage, species richness and evenness is consistent with ecophysiological theories, i.e., the acclimation potential of respiration might be higher than that of photosynthesis (Brown et al., 2004; Sullivan et al., 2020; Cabon et al., 2022). Also, thermal resistance and resilience are probably due to a combination of individual acclimation and plasticity as well as differences in species-specific responses to climate which could lead to shifts in community composition due to variations in demographic rates or plant coverage through species shift at high temperatures (Ulrich et al., 2014; Butterfield, 2015; Berdugo et al., 2019).

Importantly, we also found that plant coverage was the top regulator of AGB, and even the direct effect was stronger than temperature and precipitation, confirming again that plant coverage is the best indicator of AGB in grasslands worldwide (Ji et al., 2009; Sanaei et al., 2018a). It is not surprising that the observed direct effect of species richness was weak positive as it has been reported by many studies across the globe, indicating that species richness alone is not the best predictor of AGB which is even contrary to the predictions of the niche complementarity effect (van der Plas, 2019). As such, we found that species evenness declined AGB better than species richness, indicating again that certain species could adapt to thermal resistance. However, the positive associations among plant coverage, species richness and evenness, and their positive responses to precipitation indicate that niche complementarity plays a role in enhancing AGB in grassland communities under favourable climatic conditions. However, the negative effect of species evenness suggests that the selection effect cannot be teased apart from the niche complementarity effect in regulating AGB when temperature sensitivity is modulating the response of grassland communities. The negative effect of species evenness on AGB is likely because community AGB is determined primarily by dominant species (Smith and Knapp, 2003), i.e., *L. chinensis* is a dominant species, accounting for as much as 90% of total AGB in some sites. Moreover, *L. chinensis* is a clonal plant, likely giving it an advantage in terms of resource capture because individuals of *L. chinensis* are also usually higher than other species in the community, which further reduces evenness and increases AGB. However, if this is the situation, then *L. chinensis* is sensitive to higher temperatures but needs higher water availability for high plant coverage and AGB, which requires further experimental investigations. In addition, although we found that temperature and precipitation regulated AGB directly and indirectly *via* plant coverage and species diversity, they were positively correlated. Thus, further studies should also focus on the long-term observations of grassland communities in response to climate change factors such as temperature and precipitation seasonality as well as soil nutrients availability and heterogeneity.

## Conclusions

We show that plant coverage, as compared to species richness and evenness, plays a key role in maintaining the AGB of grasslands in northern China. As such, plant coverage promotes the coexistence of species but depends greatly on precipitation and temperature. Thus, we show that the niche complementarity and selection effects are playing a joint role in determining the AGB of grasslands. In sum, our results highlight that precipitation and temperature are two key climatic drivers of species richness, evenness, plant coverage and AGB through complex direct and indirect pathways. Our study suggests that grasslands are sensitive to climate change, i.e., a decline in water availability and an increase in atmospheric heat. We argue that plant coverage and climate change drivers related to precipitation and temperature should be included in ecological models for predicting the performance and stability of grassland communities.

## Data availability statement

The raw data supporting the conclusions of this article will be made available by the authors, without undue reservation.

## Author contributions

ZY:Conceptualization, methodology, software, validation, formal analysis, investigation, data curation, writing - original draft. YX: Data curation, investigation. LY: Investigation. LZ: Investigation, supervision, resources, project administration, writing - review & editing. AA: Writing - review & editing. All authors contributed to the article and approved the submitted version.

## Funding

This work was supported by Research on the Second Comprehensive Scientific Expedition to the Qinghai-Tibet Plateau (Grant No. 2019QZKK0301), the Science and Technology Planning Project of Inner Mongolia Autonomous Region (Grant No. 2021GG0405), the National Science and Technology Fundamental Work Special Key Special Project Editing and Researching of “Chinese Vegetation” (Grant No. 2015FY210200-24), the National Natural Science Foundation of China (Grant No. 31670532), the National Natural Science Foundation of China (Grant No. 42177347), and the National Natural Science Foundation of Inner Mongolia (Grant No. 2019MS03026). AA is supported by the special project of Hebei University (Grant No. 521100221033).

## Acknowledgments

The authors would like to thank Dr. Ma Wenhong and Dr. Feng Gang for their insightful comments on the earlier version of this manuscript. We are highly grateful to the Associate Editor (Dr. Paolo Giordani), Dr. Chang Bae-Lee and three reviewers for their insightful comments which greatly improved an earlier version of this manuscript.

## Conflict of interest

The authors declare that the research was conducted in the absence of any commercial or financial relationships that could be construed as a potential conflict of interest.

## Publisher’s note

All claims expressed in this article are solely those of the authors and do not necessarily represent those of their affiliated organizations, or those of the publisher, the editors and the reviewers. Any product that may be evaluated in this article, or claim that may be made by its manufacturer, is not guaranteed or endorsed by the publisher.
