# Supplementary material for: Precipitation and temperature regulate species diversity, plant coverage and aboveground biomass through opposing mechanisms in large-scale grasslands
Source: Front Plant Sci. 2022 Dec 1;13:999636. doi: 10.3389/fpls.2022.999636 (PMC9751382; doi:10.3389/fpls.2022.999636)
Supplement: Supplementary file 1 [file DataSheet_1.docx]

**Appendix A: Supplementary information**

**Precipitation and temperature regulate species diversity, plant coverage and aboveground biomass through opposing mechanisms in large-scale grasslands**

**Zhenyu Yao^1,2^, Yue Xin^1^, Liu Yang^3^, Liqing Zhao^1*^, Arshad Ali^4*^**

^1^Inner Mongolia Key Laboratory of Grassland Ecology and School of Ecology and Environment, Inner Mongolia University, Hohhot, 010021, China

^2^Yinshanbeilu Grassland Eco-hydrological National Observation and Research Station, China Institute of Water Resources and Hydropower Research, Beijing 100038, China

^3^ Inner Mongolia Geological Exploration Institute of Sinochem General Administration of Geology and mining, Hohhot 010020, China

^4^Forest Ecology Research Group, College of Life Sciences, Hebei University, Baoding, 071002, Hebei, China

*Corresponding authors

**Liqing Zhao**

Inner Mongolia University, Hohhot, 010021, China.

Email address: zhaotieniu@126.com

**Arshad Ali**

Forest Ecology Research Group, College of Life Sciences, Hebei University, Baoding, 071002, Hebei, China

Email addresses: [arshadforester@gmail.com /](mailto:arshadforester@gmail.com%20/) [arshadforester@hbu.edu.cn](mailto:arshadforester@hbu.edu.cn)

ORCID: 0000-0001-9966-2917

**Table S1.** Descriptive statistics of the variables across 123 meta-sites (i.e., the mean value of 3 plots per site) of pooled and three types of grasslands dominated by *Leymus chinensis* in northern China.

| Variable | Unit | Mean | S.D. | Min. | Max. |
| --- | --- | --- | --- | --- | --- |
| ***Pooled data (n = 123)*** |  |  |  |  |  |
| Latitude | Decimal degrees | 44.19 | 3.06 | 38.95 | 50.53 |
| Longitude | Decimal degrees | 116.39 | 3.86 | 109.62 | 128.22 |
| Altitude | m | 1107.89 | 426.85 | 129.40 | 2023.00 |
| Slope | ° | 5.20 | 3.91 | 0.34 | 23.65 |
| Mean annual precipitation (MAP | mm | 347.54 | 67.69 | 209.00 | 498.00 |
| Mean annual temperature (MAT) | °C | 1.66 | 2.42 | -2.90 | 7.90 |
| Species richness (S) | # | 24.28 | 9.03 | 6.00 | 54.00 |
| Species evenness (J) | # | 0.74 | 0.07 | 0.53 | 0.88 |
| Plant coverage (C) | % | 36.40 | 12.33 | 17.00 | 85.00 |
| Aboveground biomass (AGB) | g m^-2^ | 160.89 | 71.18 | 28.50 | 490.79 |
| ***Meadow data (n = 12)*** |  |  |  |  |  |
| Latitude | Decimal degrees | 45.01 | 1.58 | 42.60 | 46.83 |
| Longitude | Decimal degrees | 122.07 | 3.61 | 115.83 | 125.28 |
| Altitude | m | 471.01 | 495.01 | 129.40 | 1305.20 |
| Slope | ° | 3.33 | 2.57 | 0.34 | 9.06 |
| Mean annual precipitation (MAP | mm | 420.25 | 51.27 | 338.00 | 498.00 |
| Mean annual temperature (MAT) | °C | 3.48 | 1.91 | 0.20 | 6.60 |
| Species richness (S) | # | 21.67 | 4.56 | 14.00 | 31.00 |
| Species evenness (J) | # | 0.65 | 0.07 | 0.53 | 0.79 |
| Plant coverage (C) | % | 42.75 | 8.67 | 31.67 | 56.00 |
| Aboveground biomass (AGB) | g m^-2^ | 249.84 | 92.57 | 134.14 | 490.79 |
| ***Meadow steppe data (n = 23)*** |  |  |  |  |  |
| Latitude | Decimal degrees | 46.54 | 3.41 | 40.83 | 50.53 |
| Longitude | Decimal degrees | 118.17 | 2.57 | 112.23 | 120.39 |
| Altitude | m | 1016.68 | 460.99 | 530.00 | 2023.00 |
| Slope | ° | 6.07 | 5.22 | 0.75 | 23.65 |
| Mean annual precipitation (MAP | mm | 386.09 | 25.75 | 349.00 | 445.00 |
| Mean annual temperature (MAT) | °C | -0.57 | 1.95 | -2.90 | 6.10 |
| Species richness (S) | # | 37.61 | 7.33 | 22.00 | 54.00 |
| Species evenness (J) | # | 0.81 | 0.05 | 0.68 | 0.88 |
| Plant coverage (C) | % | 45.58 | 9.84 | 24.33 | 70.00 |
| Aboveground biomass (AGB) | g m^-2^ | 197.26 | 45.04 | 107.56 | 274.59 |
| ***Typical steppe data (n = 88)*** |  |  |  |  |  |
| Latitude | Decimal degrees | 43.46 | 2.78 | 38.95 | 49.98 |
| Longitude | Decimal degrees | 115.15 | 3.28 | 109.62 | 128.22 |
| Altitude | m | 1218.58 | 316.62 | 143.80 | 1888.00 |
| Slope | ° | 5.23 | 3.61 | 0.95 | 17.42 |
| Mean annual precipitation (MAP | mm | 327.55 | 66.33 | 209.00 | 471.00 |
| Mean annual temperature (MAT) | °C | 1.99 | 2.22 | -1.40 | 7.90 |
| Species richness (S) | # | 21.15 | 6.35 | 6.00 | 38.00 |
| Species evenness (J) | # | 0.73 | 0.06 | 0.59 | 0.85 |
| Plant coverage (C) | % | 33.13 | 11.85 | 17.00 | 85.00 |
| Aboveground biomass (AGB) | g m^-2^ | 139.25 | 59.96 | 28.50 | 276.48 |

Note: A summary of original data is presented here, whereas standardized data were used in the structural equation model, bivariate and Pearson’s correlations.

**Table S2.** Descriptive information and list of studied species in studied grasslands dominated by *Leymus chinensis* in northern China. See attached Excel sheet.

**Table S3.** The summary of the structural equation model (SEM) for linking mean annual temperature, mean annual precipitation, topographic slope, plant coverage, species richness, evenness and AGB of grassland communities dominated by *Leymus chinensis* in northern China. Significant effects (*P* < 0.05) are indicated in bold. SEM is provided in Fig. 4a. See Table S1 for abbreviations.

| Response variables | Effect | Mediator variables | Predictor variables | Path label | Beta | S.E | ci.lower | ci.upper | z-value | *P*-value |
| --- | --- | --- | --- | --- | --- | --- | --- | --- | --- | --- |
| C | Correlation |  | J | ~~ | 0.00 | 0.06 | -0.12 | 0.12 | 0.02 | 0.99 |
| C | Correlation |  | S | ~~ | 0.08 | 0.06 | -0.06 | 0.16 | 0.91 | 0.36 |
| S | Correlation |  | J | ~~ | 0.48 | 0.06 | 0.18 | 0.43 | 4.78 | **<0.001** |
| AGB | Direct |  | Slope | a | 0.16 | 0.07 | 0.02 | 0.29 | 2.32 | **0.02** |
| AGB | Direct |  | MAP | b | 0.37 | 0.08 | 0.21 | 0.53 | 4.52 | **<0.001** |
| AGB | Direct |  | MAT | b1 | -0.09 | 0.09 | -0.26 | 0.08 | -1.06 | 0.29 |
| AGB | Direct |  | C | c | 0.41 | 0.08 | 0.26 | 0.56 | 5.36 | **<0.001** |
| AGB | Direct |  | S | d | -0.02 | 0.09 | -0.20 | 0.17 | -0.18 | 0.86 |
| AGB | Direct |  | J | e | -0.18 | 0.09 | -0.35 | -0.01 | -2.11 | **0.03** |
| MAP | Direct |  | Slope | f | 0.27 | 0.09 | 0.10 | 0.44 | 3.11 | **<0.001** |
| C | Direct |  | Slope | g | 0.03 | 0.08 | -0.13 | 0.19 | 0.37 | 0.71 |
| S | Direct |  | Slope | h | 0.12 | 0.07 | -0.02 | 0.27 | 1.64 | 0.10 |
| J | Direct |  | Slope | l | 0.11 | 0.08 | -0.05 | 0.27 | 1.34 | 0.18 |
| C | Direct |  | MAP | i | 0.46 | 0.08 | 0.32 | 0.62 | 6.04 | **<0.001** |
| S | Direct |  | MAP | j | 0.36 | 0.07 | 0.23 | 0.51 | 5.13 | **<0.001** |
| J | Direct |  | MAP | k | -0.07 | 0.08 | -0.22 | 0.08 | -0.92 | 0.36 |
| MAT | Direct |  | Slope | f1 | 0.24 | 0.09 | 0.07 | 0.41 | 2.77 | **0.01** |
| C | Direct |  | MAT | i1 | -0.39 | 0.08 | -0.55 | -0.25 | -5.18 | **<0.001** |
| S | Direct |  | MAT | j1 | -0.56 | 0.07 | -0.71 | -0.43 | -7.92 | **<0.001** |
| J | Direct |  | MAT | k1 | -0.56 | 0.08 | -0.71 | -0.40 | -7.19 | **<0.001** |
| AGB | Indirect | MAP | Slope | fb | 0.10 | 0.04 | 0.02 | 0.18 | 2.56 | **0.01** |
| AGB | Indirect | C | Slope | gc | 0.01 | 0.03 | -0.05 | 0.08 | 0.37 | 0.71 |
| AGB | Indirect | S | Slope | hd | 0.00 | 0.01 | -0.02 | 0.02 | -0.18 | 0.86 |
| AGB | Indirect | J | Slope | le | -0.02 | 0.02 | -0.05 | 0.01 | -1.13 | 0.26 |
| AGB | Indirect | C | MAP | ic | 0.19 | 0.05 | 0.10 | 0.29 | 4.01 | **<0.001** |
| AGB | Indirect | S | MAP | jd | -0.01 | 0.03 | -0.07 | 0.06 | -0.18 | 0.86 |
| AGB | Indirect | J | MAP | ek | 0.01 | 0.02 | -0.02 | 0.04 | 0.84 | 0.40 |
| AGB | Indirect | MAT | Slope | f1b1 | -0.02 | 0.02 | -0.07 | 0.02 | -0.99 | 0.32 |
| AGB | Indirect | C | MAT | i1c | -0.16 | 0.04 | -0.25 | -0.08 | -3.73 | **<0.001** |
| AGB | Indirect | S | MAT | j1d | 0.01 | 0.05 | -0.09 | 0.11 | 0.18 | 0.86 |
| AGB | Indirect | J | MAT | ek1 | 0.10 | 0.05 | 0.00 | 0.20 | 2.03 | **0.04** |
| AGB | Indirect | MAT, MAP, S, J, C | Slope | (f*b)+(g*c)+(h*d)+(l*e)+(f1*b1) | 0.07 | 0.06 | -0.05 | 0.18 | 1.18 | 0.24 |
| AGB | Indirect | S, J, C | MAP | (i*c)+(j*d)+(e*k) | 0.20 | 0.06 | 0.08 | 0.32 | 3.27 | **0.001** |
| AGB | Indirect | S, J, C | MAT | (i1*c)+(j1*d)+(e*k1) | -0.05 | 0.07 | -0.19 | 0.08 | -0.77 | 0.44 |
| Slope | Total | Direct+Indirect | Slope | a+(f*b)+(g*c)+(h*d)+(l*e)+(f1*b1) | 0.22 | 0.08 | 0.07 | 0.38 | 2.80 | **0.005** |
| MAP | Total | Direct+Indirect | MAP | b+(i*c)+(j*d)+(e*k) | 0.56 | 0.07 | 0.42 | 0.71 | 7.68 | **<0.001** |
| MAT | Total | Direct+Indirect | MAT | b1+(i1*c)+(j1*d)+(e*k1) | -0.14 | 0.07 | -0.29 | 0.00 | -1.98 | **0.04** |


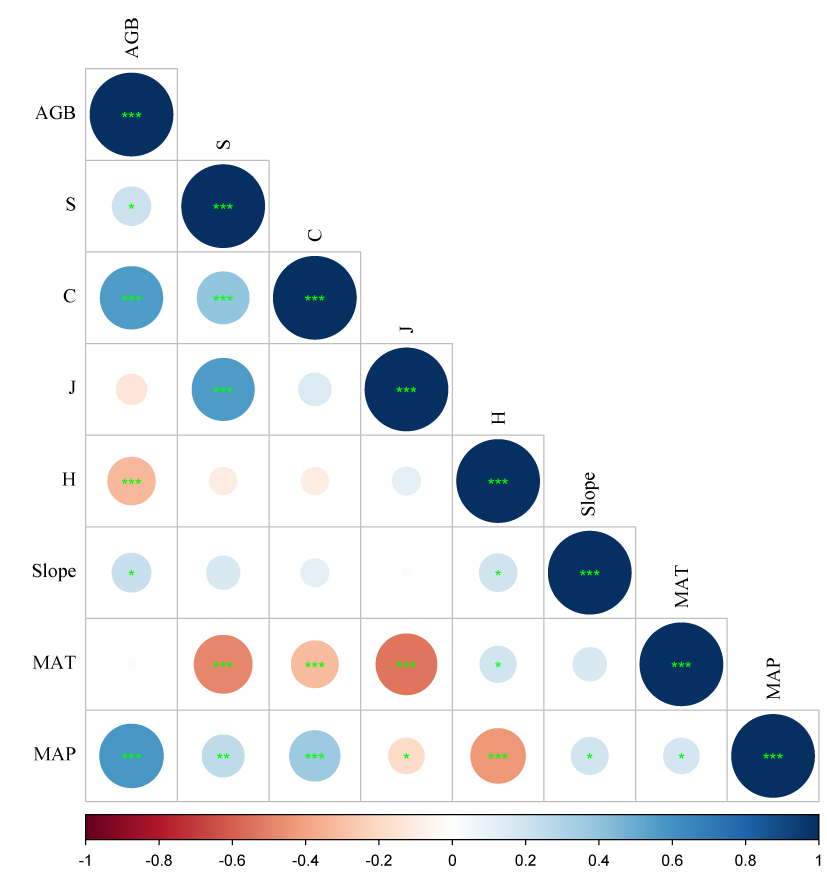


**Figure S1** The Pearson’s correlation coefficients matrix for tested variables used in the SEMs for linking climatic factors (either MAP or MAT), topographic slope, plant coverage, species richness, evenness and AGB of *Leymus chinensis* communities in northern China. Dark red to dark blue colors represent negative to positive correlations, with ****, *P* < 0.001; **, *P* < 0.01; *, *P* < 0.05. See Table S1 for abbreviations.
